# Preparation and Characterization of Dual-Stabilized Vanillin Complexes Based on Soy Protein Isolate Through pH-Shifting Strategy

**DOI:** 10.3390/foods15071240

**Published:** 2026-04-05

**Authors:** Xudong Wang, Kaiwen Wu, Yating Shen, Zhenglin Wu, Weijian Yuan, Weina Wu, Fengping Yi

**Affiliations:** School of Perfume and Aroma Technology, Shanghai Institute of Technology, Shanghai 201418, China

**Keywords:** soy protein isolate, vanillin, nano-complex, pH-shifting treatment, thermal stability

## Abstract

Vanillin is widely used in foods, but its poor water dispersibility and limited stability reduce its flavor performance during processing and storage. In this study, soy protein isolate (SPI) was used as a food-grade carrier to prepare soy protein isolate–vanillin (SPIV) complexes via a pH-shifting strategy. SPI and vanillin were first adjusted to pH 9.0, where SPI unfolded and vanillin was deprotonated and dispersed in the solution and then readjusted to pH 7.0 to form SPIV complexes. Vanillin was incorporated into SPI at different loading levels of 0.5, 1.0, 2.5, and 5.0 mg/mL, corresponding to 9–50 wt.% relative to SPI. The binding efficiency of vanillin decreased from 91.03 wt.% to 69.43 wt.% with increasing vanillin loading. Moderate loading preserved the globular morphology of SPI, whereas excessive loading (≥33.33 wt.%) induced vanillin nanocrystal formation and aggregation. Spectroscopic analyses and molecular docking indicated that vanillin interacted with soy proteins through a combination of covalent and noncovalent interactions. Compared with free vanillin, SPIV showed improved color, light, and thermal stability. Among the tested samples, SPIV2 exhibited the most favorable interfacial behavior and application performance, producing more stable emulsions and higher flavor scores in simplified beverage and soy milk models. These findings establish a loading-dependent structure–function relationship in SPIV complexes and provide practical guidance for the design of soy protein-based carriers for flavor stabilization and delivery.

## 1. Introduction

Soy protein isolate (SPI) is a high-purity plant-derived protein sourced from soybean meal, a byproduct of the soy oil processing industry, which endows it with exceptional supply availability and sustainable economic value [1]. Beyond its cost-effective and renewable advantages, SPI features a well-balanced essential amino acid profile, moderate amphiphilicity and inherent lipid-stabilizing capacity [2]. The abundant amino acid residues provide multiple reactive sites that can be chemically modified to tune and improve its physical and chemical properties [3]. Previous studies have validated that bioactive molecules can form complexes with SPI through either covalent or noncovalent interactions [4]. For example, typical polyphenolic compounds including gallic acid, tea polyphenols and anthocyanins have been demonstrated to bind with SPI via both covalent and noncovalent pathways, forming functional complexes with enhanced performance [5,6,7]. Notably, these SPI-polyphenol complexes not only improve the chemical stability and bioavailability of the bound polyphenols but also exhibit superior gelation properties and enhanced emulsion droplet stabilization capacity compared to native SPI, expanding its practical application scope in food systems [8,9].

Most food-grade flavor compounds are inherently hydrophobic, thermolabile, and susceptible to oxidative degradation or volatilization during food processing and long-term storage. Consequently, flavored food products frequently suffer from significant flavor loss, compromised sensory quality, shortened shelf life, and reduced consumer acceptability, representing a persistent technical challenge in the flavor and food manufacturing industry [10]. Encapsulation of hydrophobic flavor compounds has emerged as a well-established and effective strategy to enhance their aqueous dispersibility, chemical stability, and controlled release behavior in complex food matrices [11]. These flavor compounds can be encapsulated in polymeric and nano/microscale materials in the form of molecules, nanocrystals, or oil droplets, thereby forming complex systems. Approaches based on carrier-assisted stabilization have therefore been widely explored to improve their dispersibility, stability, and retention in food systems. A wide range of inorganic, organic, and organic–inorganic hybrid materials have been investigated for this purpose [12,13]. In recent years, many bio-based macromolecules have also attracted increasing attention because of their high biocompatibility, good degradability, and broad availability. These materials can interact with flavor compounds through adsorption, association, or complex formation, thereby improving their stability and performance in food matrices. Cyclodextrins and starch are commonly used flavor carriers in the food industry and are generally recognized as safe (GRAS). Their low cost and simple processing make them attractive to flavor manufacturers [14]. However, these systems often suffer from relatively low encapsulation efficiencies, which increases the amount of carrier required per unit of flavor and thereby raises formulation costs. In addition, the uncontrolled release of flavorings during storage limits the extent to which these carriers can actually improve flavor stability in real applications. To address these limitations, a variety of carriers with controlled release properties have been developed to improve the stability and release behavior of flavorings in different food matrices. For example, engineered mesoporous silica nanoparticles with tailored pore sizes and compositions can efficiently adsorb flavor molecules [15]. By fine-tuning their chemical environment and microstructure, these particles enable controlled and rapid release under specific light or pH conditions [16]. Another strategy is to assemble supramolecular aggregates, such as metal–organic frameworks, which allow slow release of flavorings under neutral to alkaline conditions and rapid release under acidic conditions [17,18]. These material-based approaches provide valuable insights into enhancing flavor stability and persistence in food systems. However, the structural complexity of the carriers and the multi-step preparation procedures make scale-up from laboratory to industrial production challenging.

Vanillin is one of the most iconic and widely consumed food flavoring agents. characterized by a distinct creamy, sweet and aromatic vanilla scent that serves as the key compound of vanilla extract. As a result, vanillin is widely used in a broad range of food products, including beverages, baked goods and dairy products such as ice cream, with global market demand continuing to rise steadily [19]. However, vanillin is poorly dispersible in water and is sensitive to heat. Its phenolic aldehyde structure also makes it prone to discoloration during processing and storage, which can alter the sensory profile of vanilla-flavored products and poses a challenge for food processing and shelf-life management [20]. In current flavor applications, vanilla flavor formulations rely on ethanol or synthetic surfactants to improve vanillin dispersibility, but with the growing global demand for clean-label and naturally derived foods, there is increasing interest in replacing part of these surfactants with food-grade macromolecules from natural sources. Soy protein isolate (SPI) has been shown to form stable complexes with phenolic compounds via synergistic covalent and noncovalent interactions [21]. This concept may also be feasibly extended to monophenolic molecules. Vanillin contains both a phenolic hydroxyl group and an aldehyde group, providing multiple potential interaction modes with SPI, including covalent bonding and diverse noncovalent contacts [22]. SPI is therefore a promising carrier to improve the dispersibility and stability of vanillin in water. Nevertheless, studies that use SPI alone to stabilize vanillin are still scarce.

Herein, we propose that SPI, a byproduct of soy oil processing, can serve as a carrier for vanillin to improve its dispersibility and stability. Vanillin at different loadings was incorporated into SPI via a pH-shifting strategy to obtain a series of SPIV complexes. Changes in microstructure and chemical signatures were analyzed to probe potential concentration-dependent interactions between vanillin and SPI, and molecular docking was used to identify their binding sites. The functional properties of these complexes were then evaluated, including antioxidant activity, heat stability and emulsifying performance. This work not only identifies an appropriate SPI-to-vanillin ratio for use as a carrier system but also delineates how the structural and functional properties of SPIV evolve with vanillin content. We expect that these findings will provide useful guidance for the design of flavor stability systems in food applications.

## 2. Materials and Methods

### 2.1. Materials

Soy protein was obtained from Archer-Daniels-Midland Co. (Chicago, IL, USA), and soy protein isolate (SPI, 7S Mw ~180 kDa and 11S Mw ~320–360 kDa) was prepared by a conventional pH-shifting method as described in a previous report [5]. Vanillin (99%, Mw = 152.15 Da) and potassium bromide (KBr, SP) were purchased from Macklin Biochemical Co., Ltd. (Shanghai, China). PBS (0.01 M, pH 7.2–7.4) was bought from Beijing Solarbio Science & Technology Co., Ltd. (Beijing, China). Medium-chain triglyceride (MCT) was bought from Shanghai Yuanye Bio-Technology Co., Ltd. (Shanghai, China). 1,1-Diphenyl-2-picrylhydrazyl (DPPH) and 2,2′-azino-bis(3-ethylbenzothiazoline-6-sulfonic acid (ABTS) were purchased from Sigma-Aldrich Co. (St. Louis, MO, USA). Food-grade sugar, citric acid, sodium citrate, sodium bicarbonate, and sodium benzoate were bought from Jiahexuri Food Additive Co., Ltd. (Zhengzhou, China). Deionized (DI) water was used throughout the experiment.

### 2.2. Preparation of SPIV Complex

For SPI dispersion, 10 mg/mL SPI was dissolved in DI water, stirred for 2 h, and stored overnight at 4 °C. For vanillin solution, 10 mg/mL vanillin and 1.25 mg/mL NaOH were dissolved in DI water, heated at 50 °C for 10 min with an ultrasonic waterbath (40 kHz, Kunshan Ultrasonic Instrument Co., Kunshan, China). Both SPI and vanillin solution were adjusted to pH 9 by 2 M NaOH, stirred for 5 min, and then mixed at different volume ratios (from 10:1 to 1:1), stirred for 30 min, and then quickly adjusted to pH 7.0 with 1 M HCl. A portion of SPIV was freeze-dried and stored at −20 °C for subsequent experiments. SPIV dispersions were stored at 4 °C for no longer than 3 days. Therefore, SPIV complexes containing 9–50 wt.% vanillin were prepared and designated as SPIV1 (9 wt.% Vanillin), SPIV2 (16.7 wt.% Vanillin), SPIV5 (33.3 wt.% Vanillin), and SPIV10 (50 wt.% Vanillin). These vanillin contents could be used to understand the SPIV complex formation and performance at high vanillin loadings.

### 2.3. Binding Ratio of Vanillin

The determination of free vanillin content and vanillin binding efficiency was based on previous research with slight modifications [23]. Briefly, 1 mL of freshly prepared SPIV complex dispersion (SPI at 10 mg/mL) was loaded into a dialysis bag (3.5 kDa Mw) and dialyzed against 100 mL of deionized water for 24 h to remove unbound vanillin. The absorbance of the dialysate was measured at 346 nm using a SpectraMax iD3 microplate reader (Molecular Devices, San Jose, CA, USA), and both the free vanillin content and vanillin binding efficiency were calculated using a vanillin standard curve.

### 2.4. Particle Size and ζ-Potential

The particle size and ζ-potential of SPI and SPIV complexes were measured using a Zetasizer Pro (Malvern Instruments, Worcestershire, UK). Before measurement, the SPI and SPIV dispersions were diluted with PBS (0.01 M, pH 7.4) to an SPI concentration of 1 mg/mL and filtered through a 0.45 μm membrane to remove large aggregates.

### 2.5. Transmission Electron Microscopy (TEM)

Transmission electron microscopy (TEM) was used to examine the effect of vanillin concentration on the structure of SPI. SPI and SPIV complex dispersions diluted with deionized water were dropped onto copper grids. After sufficient deposition, excess water was removed. The microstructural changes in SPI and SPIV were then observed using a JEM-1400 microscope (JEOL, Tokyo, Japan) operated at 100 kV. To observe possible microstructural changes in the protein, uranyl acetate staining was not used in this study, following the procedure adopted in a previous report [5].

### 2.6. Scanning Electron Microscopy (SEM)

The microstructure of freeze-dried samples was observed by scanning electron microscopy (SEM, SU8020, Hitachi, Tokyo, Japan). Samples were gently sprinkled onto the surface of conductive tape, and excess powder was removed by gentle air blowing. The sample surfaces were then sputter-coated with gold to improve conductivity.

### 2.7. Thermogravimetric Analysis (TGA)

Thermal analysis of freeze-dried SPI, vanillin and SPIV complexes was performed using an STA6000 analyzer (PerkinElmer Inc., Shelton, CT, USA). Samples were heated from 30 to 600 °C at a rate of 10 °C/min under a high-purity nitrogen atmosphere.

### 2.8. Water Contact Angle

Water contact angles were measured using a contact angle goniometer (SZ-CAMC333, Shanghai Sunzern Instrument Co., Ltd., Shanghai, China). SPI and SPIV complex dispersions were evenly drop-cast onto glass slides and dried at 30 °C to form films. A 5 μL droplet of deionized water was placed on the film surface and held for 5 s before imaging. The contact angle was then calculated by fitting the droplet profile using the Young–Laplace equation.

### 2.9. UV-Visible (Vis) and Fluorescence Spectrum

UV–Vis and fluorescence spectra were recorded using a SpectraMax iD3 microplate reader (Molecular Devices, San Jose, CA, USA). Before measurement, SPI and SPIV complex dispersions were diluted with PBS (0.01 M, pH 7.4) to the same SPI concentration. UV–Vis spectra were collected from 230 to 800 nm with a step size of 1 nm. For fluorescence measurements, excitation (Ex) wavelengths were set at 258 nm (Phe) and 275 nm (Tyr), and emission (Em) spectra were recorded from 300 to 450 nm.

### 2.10. FT-IR Spectra

Freeze-dried SPI, vanillin and SPIV complex powders were mixed with dried KBr at a mass ratio of 1:20 (*w*/*w*) and thoroughly ground. FT-IR spectra were collected using a Nicolet iS20 spectrometer (Thermo Fisher Scientific, Waltham, MA, USA). Samples were scanned over the range of 4000–400 cm^−1^ with a resolution of 4 cm^−1^.

### 2.11. Molecular Docking

The protein structures of SPI (7S and 11S) were obtained from the Protein Data Bank (PDB) with the accession codes 1OD5 (β-conglycinin) and 9IS2 (glycinin), respectively. The three-dimensional structure of vanillin was downloaded from the PubChem database and energy-minimized in Chem3D using the MMFF94 force field. Molecular docking simulations were performed with AutoDock Vina (v 1.1.2). The conformation with the highest docking score was taken as the optimal binding pose and was further visualized and analyzed in PyMOL (v 2.5).

### 2.12. Antioxidant Ability

The antioxidant activity of SPI and SPIV was evaluated based on a previous report with slight modifications [24]. For the DPPH radical scavenging assay, a DPPH stock solution (0.1 mM) was prepared by dissolving DPPH in 95 vol.% ethanol/water. A 100 μL sample was mixed with 100 μL of the DPPH stock solution and incubated in the dark at room temperature for 30 min. The absorbance at 517 nm was then measured immediately using a SpectraMax iD3 microplate reader (Molecular Devices, San Jose, CA, USA). For the ABTS radical scavenging assay, an ABTS stock solution was prepared by mixing 7.4 mM ABTS with 4.9 mM potassium persulfate at a 1:1 volume ratio and incubating the mixture in the dark for 12 h. Then, 20 μL of the sample was mixed with 180 μL of the ABTS stock solution and incubated in the dark at room temperature for 6 min. The absorbance at 734 nm was immediately measured using the same microplate reader.

### 2.13. Stability of Vanillin

To evaluate the color stability of vanillin, freshly prepared SPIV1, SPIV2, SPIV5, and SPIV10, together with vanillin dispersions at the corresponding vanillin concentrations (pH 7.4, neutralized with NaOH), were kept at room temperature for 1 day. The color changes in the samples were then recorded using the rear camera of a smartphone (iPhone 13 Pro, Apple Inc., Cupertino, CA, USA). For thermal stability of vanillin, free vanillin, SPIV2 and SPIV5 were used to evaluate the thermal stability of vanillin in SPIV complexes with different microstructures. Samples were placed in separate glass dishes and heated in an open state in an oven at 40 °C to allow vanillin to volatilize. For free vanillin, the loss rate was directly determined by measuring the mass decrease after heating. For SPIV2 and SPIV5, 5 mL of ethanol was added to the samples after cooling to room temperature. The mixtures were then sonicated in an ice–water bath using an ultrasonic processor (Scientz-IID, Ningbo Scientz Biotechnology Co., Ningbo, China) equipped with a Φ3 (1/8″), 250 μm microtip to extract the retained vanillin. The absorbance of the ethanolic extracts was measured using a SpectraMax iD3 microplate reader (Molecular Devices, San Jose, CA, USA), and the vanillin content was quantified using a standard curve.

### 2.14. Preparation and Characterization of Emulsion

Oil-in-water (O/W) emulsions were prepared by adding MCT to SPI or SPIV dispersions. SPI or SPIV complexes were dispersed in deionized water at a protein concentration of 10 mg/mL and stirred for 2 h to obtain homogeneous dispersions. The dispersions were then mixed with MCT at a volume ratio of 9:1 (water/oil) and homogenized using a high-speed homogenizer at 10,000 rpm for 3 min.

### 2.15. Characterization of Emulsion

The droplet size of emulsions stabilized by SPI or SPIV complexes was measured using a Zetasizer Pro (Malvern Instruments, Worcestershire, UK). The morphology of emulsions was characterized using high-resolution fluorescence microscopy (DM6B, Leica Microsystems, Wetzlar, Germany). Fast Green and Nile Red were used to fluorescently label the protein and oil phase, respectively. The dyes were added directly to the emulsion and vortexed briefly to ensure uniform mixing. A 10 μL droplet of the stained emulsion was then placed on a glass slide and gently covered with a coverslip. Bright-field and fluorescence images were captured for further analysis.

### 2.16. Interfacial Tension

SPI, SPIV2, and SPIV5 were used to evaluate differences in interfacial performance among SPIV complexes with distinct microstructures at the oil–water interface. Interfacial tension was measured using a contact angle goniometer (OCA25, Dataphysics, Filderstadt, Germany). MCT was added to a rectangular glass cell, and 10 μL of the sample, diluted with deionized water to 1 mg/mL based on SPI concentration, was injected into the MCT phase using a 1 mL syringe while keeping the needle and droplet suspended in the oil phase. The interfacial tension was calculated by fitting the droplet profile with the Young–Laplace equation, and the values recorded over 600 s were used for subsequent analysis.

### 2.17. Sensory Evaluation

Vanillin at a concentration of 0.5 mg/mL, either dissolved in ethanol or incorporated into SPIV2 complexes, was added to simplified soft drink and soy milk models. The soft drink model contained 6 wt.% sugar, 0.5 wt.% citric acid, and 0.3 wt.% sodium citrate, whereas the soy milk model contained 3 wt.% soy protein concentrate, 3 wt.% sugar, 0.5 wt.% sodium bicarbonate, and 0.5 wt.% sodium benzoate. All samples were pasteurized (heated to 80 °C, and then stored for 1 min) once and then rapidly cooled to room temperature. One sample was stored at 4 °C as a control, while the remaining samples were stored at 40 °C for 2 weeks for accelerated aging based on previous research [21]. After 2 weeks, the samples were removed and rapidly cooled again to room temperature. Following previously reported methods, twenty trained panelists evaluated the aroma of randomly coded samples. A nine-point hedonic scale was used to assess changes in flavor, where a score of 1 indicated that the aroma had almost disappeared, differed completely from the reference, or exhibited off-odors, and where a score of 9 indicated that the aroma was almost identical to the reference. All participants provided written informed consent before the sensory evaluation.

### 2.18. Statistical Analysis

All experiments were conducted at least three times. The average result was expressed as the mean ± standard deviation (SD) unless otherwise specified. The data were analyzed by the one-way analysis of variance (ANOVA). Means were separated by Tukey’s multiple range test when ANOVA was significant (*p* < 0.05) by SPSS Statistics 26 (SPSS Inc., Chicago, IL, USA).

## 3. Results and Discussion

### 3.1. Preparation of Soy Protein Isolate-Vanillin (SPIV) Complex

In this study, a facile pH-shifting strategy was employed to load vanillin into the SPI matrix and fabricate stable SPIV complexes, and this fabrication protocol was optimized based on the structural responsiveness of SPI under alkaline conditions. Briefly, native SPI dispersion and vanillin solution were individually adjusted to pH 9.0 prior to mixing, which is a critical step for successful complex assembly. According to previous reports, under this condition, the protein structure of SPI unfolds, while vanillin is deprotonated and dispersed in the aqueous phase in a molecular form. After thorough mixing of the alkaline SPI dispersion and vanillin solution, the system pH was rapidly adjusted back to neutral 7.0, inducing the refolding of unfolded SPI chains. During this dynamic unfolding-refolding process, vanillin molecules were efficiently encapsulated and immobilized within the SPI three-dimensional network via non-covalent and potential covalent interactions, ultimately forming stable SPIV complexes. Previous studies have shown that curcumin and eugenol were efficiently loaded into SPI through a pH-shifting approach, thereby improving their stability and bioaccessibility [25,26].

**Figure 1:**
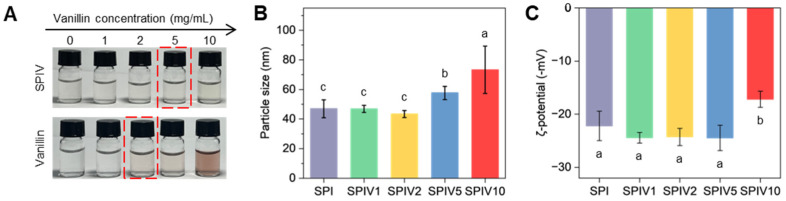
Characterization of SPIV with different vanillin concentrations. (**A**) Photographs of SPIV and vanillin dispersions stored at room temperature for 1 day. The red box marks the vanillin concentration at which a visible color change can be observed by the naked eye. (**B**) Particle size of freshly prepared SPI and SPIV. Values with different superscript letters are significantly different (*p* < 0.05). (**C**) ζ-potential of freshly prepared SPI and SPIV. Values with different superscript letters are significantly different (*p* < 0.05).

The macroscopic appearance stability of the prepared SPIV complexes was evaluated after storage at room temperature for 24 h, and the results are presented in Figure 1A. All SPIV samples remained relatively colorless and transparent. In contrast, vanillin aqueous solutions with concentrations ranging from 1.67 mg/mL to 2.5 mg/mL exhibited distinct browning and color deepening after identical storage conditions. This significant macroscopic difference clearly demonstrates that, at relatively high vanillin loadings, SPIV complexes formed by the pH-shifting method effectively suppressed the color change in vanillin. Because vanillin contains both a phenolic hydroxyl group and an aldehyde group, it is prone to oxidation and the formation of quinonoid structures, which is accompanied by color darkening. Previous studies have suggested that vanillin can act as a molecular adhesive to improve the properties of protein particles [27,28]. However, studies on how vanillin content affects the formation and microstructure of protein particles remain limited.

As shown in Table 1, as the vanillin concentration added during preparation increased sequentially from 0.5 to 5 mg/mL, the content of free vanillin in different SPIV systems also increased continuously from 0.045 to 1.529 mg/mL. Accordingly, the vanillin binding efficiency (bound vanillin/total vanillin) decreased significantly from 91.03 wt.% to 69.43 wt.% with the elevation of vanillin feeding level. The inverse correlation between vanillin addition concentration and binding efficiency clearly indicates that, as the feeding level increased, the amount of unbound vanillin gradually increased, suggesting that vanillin progressively approached and exceeded the available binding sites on SPI. The loading capacity of vanillin (bound vanillin/total SPI) was then calculated and was found to increase with increasing vanillin addition, reflecting the actual loading level of vanillin relative to SPI in different SPIV complexes.

Particle size remained at low vanillin loadings but increased markedly at higher vanillin loadings, especially from SPIV5 onward (Figure 1B). The particle size changes may be attributed to intermolecular crosslinking formed by vanillin bound within SPI during the pH-shifting process. When the vanillin concentration was low, crosslinking tended to occur within individual SPI. When the vanillin concentration exceeded the available binding sites, multiple SPI entities may be present. The ζ-potential showed the opposite trend. The absolute value of the ζ-potential showed only limited variation at low vanillin loadings but decreased at higher vanillin levels, especially from SPIV5 onward (Figure 1C). A small amount of vanillin enhanced the surface charge of SPI, which is consistent with previous studies on SPI–anthocyanin and SPI–EGCG systems [29,30]. However, a high level of vanillin may destroy the particle-like structure of SPI and promote aggregation, leading to a decrease in ζ-potential. Changes in ζ-potential reflect the colloidal stability of the particles. Therefore, an excess of vanillin may cause SPIV aggregation and compromise its stability.

**Figure 2:**
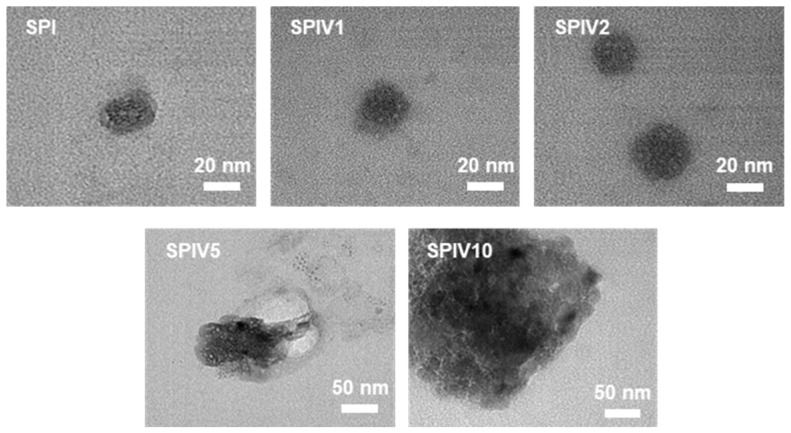
Representative microstructure of SPI or SPIV dispersion captured by TEM, the scale bar = 20 or 50 nm.

**Figure 3:**
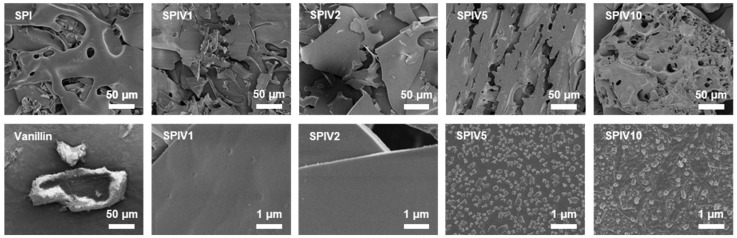
Representative microstructure of freeze-dried SPI, SPIV1, SPIV2, SPIV5, SPIV10, and vanillin, captured by SEM, the scale bar = 50 or 1 μm.

The effect of vanillin concentration on SPIV was evaluated by examining its microstructure. TEM images of SPI alone showed a spherical morphology, which is typical of globular proteins (Figure 2). At vanillin concentrations below 16.7 wt.% (SPIV1 and SPIV2), SPIV largely retained this native spherical morphology, indicating that vanillin at these levels did not markedly disrupt the intrinsic globular structure of SPI. When the vanillin concentration increased to 33.3 wt.% (SPIV5), the spherical morphology became less distinct, the particle size increased, and irregular structures appeared, suggesting that excess vanillin perturbed protein organization. When the vanillin concentration was further increased to 50.0 wt.% (SPIV10), pronounced aggregation occurred, leading to the formation of large agglomerates. In addition, SEM was used to examine the morphology of SPIV after freeze-drying (Figure 3). SPI exhibited a smooth, film-like structure characteristic of polymeric materials. When the vanillin concentration was below 16.7 wt.% (SPIV1 and SPIV2), SPIV showed a similar continuous film morphology with a smooth surface. As the vanillin concentration increased to 33.3 wt.% and 50.0 wt.%, the originally continuous film structure was damaged and the microstructure became discontinuous. At higher magnification, particle-like deposits were observed on the surface, indicating that the binding sites of SPI for vanillin were saturated and that vanillin grew as solid particles on the SPIV surface. This characteristic is consistent with the previous result for SPI–ellagic acid complex, in which ligands in excess of the available binding sites tended to accumulate on the protein surface as small aggregates [31].

**Figure 4:**
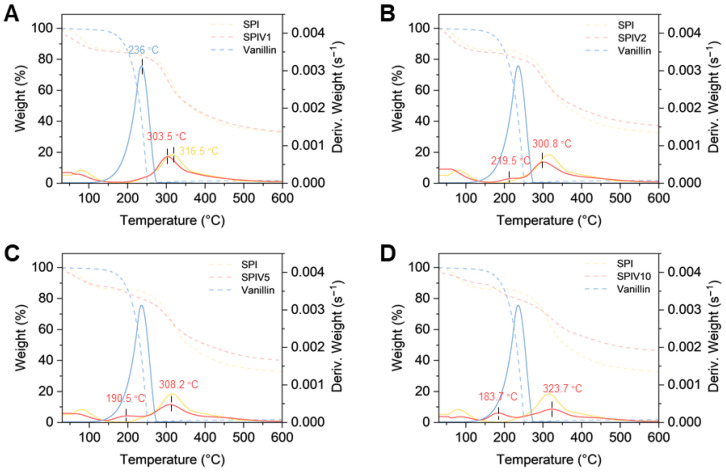
TGA and DTG curves of SPI, vanillin, and SPIV with different vanillin concentrations. (**A**) TGA and DTG curves of SPI, vanillin, and SPIV1. (**B**) TGA and DTG curves of SPIV2. (**C**) TGA and DTG curves of SPIV5. (**D**) TGA and DTG curves of SPIV10.

Thermogravimetric analysis (TGA) was conducted to verify the intermolecular interactions between SPI and vanillin within the fabricated SPIV complexes. Vanillin is a thermally sensitive small molecule compound, with a distinct thermal decomposition range of 120–272 °C, and a characteristic degradation temperature (Td) of 236 °C. Consistent with the previous literature findings, SPI showed a broader thermal decomposition range from 200 to 550 °C, with a corresponding Td of 316.5 °C [32]. SPIV1 and SPIV2 contained relatively low levels of vanillin and largely retained the native globular structure of SPI. These two samples were discussed together. SPIV1 and SPIV2 displayed thermal degradation profiles similar to that of SPI (Figure 4A,B). The characteristic degradation peak of free vanillin at 236 °C disappeared or became markedly attenuated, and the Td values shifted to 303.5 °C and 300.8 °C, respectively. These changes suggest that incorporation of vanillin into the SPI matrix altered its thermal behavior [33]. As the vanillin content increased, SPIV5 and SPIV10 exhibited two distinct thermal degradation stages, the first stage between 170 and 250 °C (Td SPIV5 = 190.5 °C, Td SPIV10 = 183.7 °C) and a second stage between 250 and 550 °C (Td SPIV5 = 308.2 °C, Td SPIV10 = 323.7 °C) (Figure 4C,D). A decrease in Td in the first stage and an increase in Td in the second stage were observed. The lower Td in the first stage may reflect the thermal loss of vanillin. Compared with larger vanillin domains, vanillin present in smaller domains can lose mass more rapidly upon heating, which could explain the reduced Td (Figure 3, vanillin vs. SPIV5 and SPIV10). The higher Td in the second stage may be due to the thermal degradation of SPI or SPIV complexes, where vanillin acts as a crosslinker and promotes aggregation of SPI. In contrast with SPIV1 and SPIV2, this aggregation generated larger and more complex assemblies, which reduced the rate of thermal mass loss and led to an increased Td.

**Figure 5:**
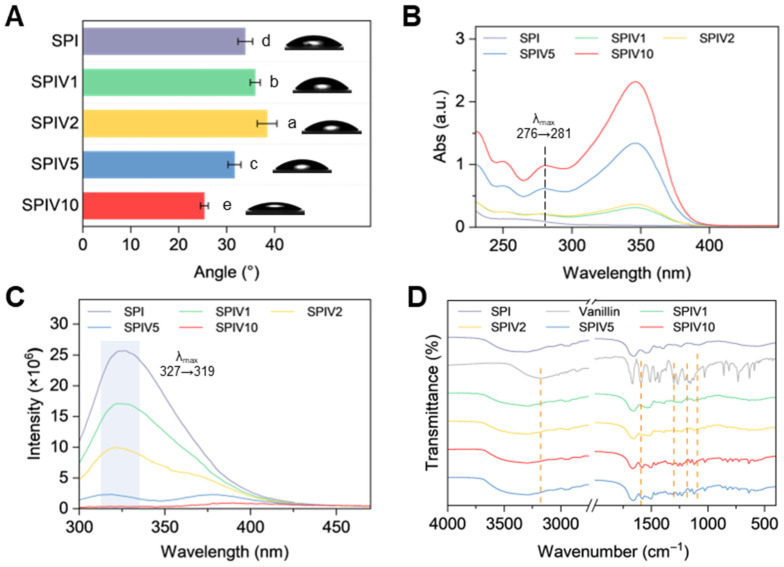
Characterization of SPI and SPIV. (**A**) Water contact angle of SPI and SPIV. (**B**) UV-vis spectra of SPI and SPIV. (**C**) Fluorescence spectra of SPI and SPIV with Ex: 258 (Phe). (**D**) FTIR spectra of SPI and SPIV. Values with different superscript letters are significantly different (*p* < 0.05).

The hydrophobicity of SPI and SPIV was also evaluated by water contact angle. As the vanillin content increased, hydrophobicity first increased and then decreased (Figure 5A). The turning point in hydrophobicity occurred at SPIV2, which is consistent with the results described above. At a lower vanillin concentration, vanillin takes part in the structure change on the single SPI by forming a crosslinked network, leading to an increase in surface hydrophobicity. When there is excess vanillin, it may accumulate on the surface or form larger aggregates, which imparts a more hydrophilic character to the complexes.

### 3.2. Chemical Structure of SPIV

The changed protein structure by vanillin was determined by Ultraviolet-visible (UV-Vis) and fluorescence spectra [6]. As the vanillin concentration increased, characteristic absorption bands appeared at 250, 280, and 360 nm (Figure 5B). The band at 280 nm showed a red shift from 276 to 281 nm. This absorption at 280 nm originates from the aromatic ring of vanillin and the conjugated double bonds of tryptophan and tyrosine residues in SPI [34]. Different vanillin concentrations kept the 280 nm characteristic peak. Therefore, this red shift can be attributed to interactions between vanillin and the *Trp* and *Tyr* residues. Subsequently, fluorescence spectroscopy was used to probe changes in the tertiary structure of SPI and the microenvironment of phenylalanine (*Phe*) residues. Fluorescence spectra of SPIV recorded at excitation wavelengths of 258 nm showed pronounced quenching as the vanillin concentration increased (Figure 5C). When the vanillin content reached 33.3 wt.%, the fluorescence was almost completely quenched. This trend was consistent with the microstructural observations and further confirmed that the binding sites on SPI were saturated at this concentration. The unbound vanillin crystallized around SPIV and persisted as small, stable particles.

FTIR spectroscopy was employed to investigate chemical interactions and functional group changes between vanillin and SPI. As shown in Figure 5D, Vanillin exhibited characteristic absorption bands in the region of 1800–1100 cm^−1^, which correspond to its aromatic ring vibrations, C-O stretching of phenolic hydroxyl groups, and the signature band of the methoxy group (Figure 5D). The aromatic ring band at 1590 cm^−1^ shifted to 1580 cm^−1^ in SPIV1-SPIV5. Since SPI does not display a distinct band at this position, this shift can be attributed to π–π stacking. In addition, the amide I region of SPI shifted from 1655 cm^−1^ to 1661–1663 cm^−1^ in SPIV, suggesting that vanillin altered the local chemical environment and conformation of SPI, which is consistent with possible covalent and noncovalent interactions between vanillin and SPI. The phenolic C-O stretching band at around 1300 cm^−1^ became weaker, which may be related to the formation of hydrogen bonds and covalent bonds during the interaction with the protein. Furthermore, at pH 7, the phenolic hydroxyl group of vanillin undergoes deprotonation to form -O-, which may also contribute to the attenuation of this band. The bands at 1155 cm^−1^ and 1125 cm^−1^ also provided evidence for hydrogen bonding and hydrophobic interactions between vanillin and SPI. Collectively, these FTIR results confirmed that the formation of SPI–vanillin complexes was driven by a combined network of covalent and noncovalent interactions.

### 3.3. Molecular Docking Between SPI and Vanillin

**Figure 6:**
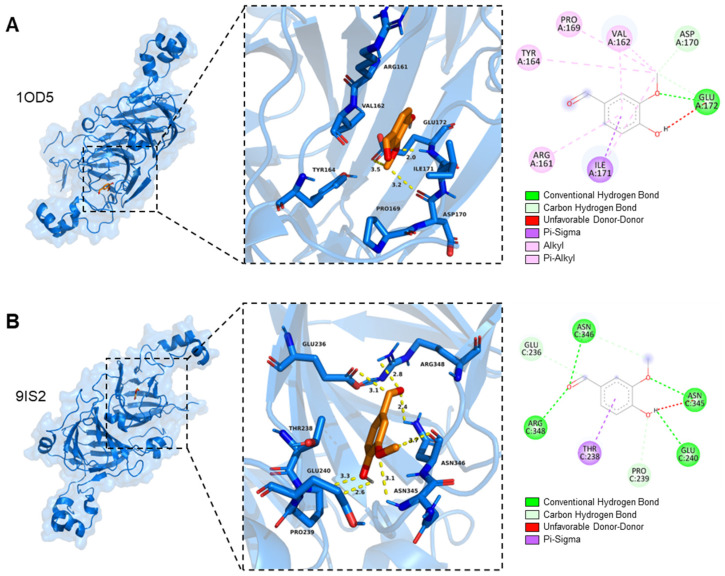
Molecular docking between vanillin and soy protein. (**A**) 1OD5 (β-conglycinin). (**B**) 9IS2 (glycinin).

Possible molecular interactions between vanillin and SPI were explored by molecular docking [28]. The best predicted docking poses showed affinities of −5.180 kcal/mol for 1OD5 and −5.755 kcal/mol for 9IS2, suggesting a favorable but moderate binding tendency of vanillin toward both soy proteins. For 1OD5, vanillin was predicted to occupy a shallow surface pocket, where it was mainly associated with a limited number of hydrogen bonds and hydrophobic contacts in a relatively open and hydrophobic environment (Figure 6A). The phenolic hydroxyl group of vanillin was predicted to interact with the side-chain carboxyl group of GLU172. In addition, the carbonyl and methoxy groups may form hydrogen bonds or polar contacts with residues such as ILE171 and PRO169. VAL162, ILE171, PRO169, and TYR164 were located around one side of the aromatic ring and may provide a hydrophobic microenvironment, while ASP170 at the rim of the pocket may contribute additional polar contacts. For 9IS2, vanillin was predicted to bind in a deeper and more polar pocket, where multiple hydrogen bonds, polar interactions, and electrostatic contacts may be involved (Figure 6B) [35]. The phenolic hydroxyl group was predicted to interact with ASN345 and ASN346, while the carbonyl and methoxy groups may establish hydrogen bonds or polar contacts with GLU236, GLU240, and THR238. GLU236, GLU240, and ARG348 may further contribute to electrostatic or dipolar interactions and reinforce the polar environment around the ligand. In addition, PRO239 may help restrict the pocket geometry and thereby stabilize the predicted ligand conformation. Taken together, the docking results provide a plausible model for the possible binding sites and interaction patterns between vanillin and soy proteins. The molecular docking results were in good agreement with the structural changes observed in SPIV. Vanillin was predicted to bind within protein pockets and interact with surrounding residues through hydrogen bonding, hydrophobic interactions, and polar contacts. At higher vanillin loadings, pronounced fluorescence quenching of amino acid residues was also observed, indicating that the interactions between vanillin and SPI involved multiple binding modes.

### 3.4. Antioxidant Ability of SPIV

**Figure 7:**
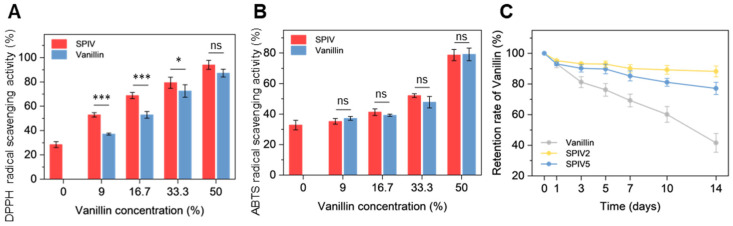
Antioxidant ability and vanillin thermal stability of SPIV. (**A**) DPPH radical scavenging activity of SPI and SPIV. (**B**) ABTS radical scavenging activity of SPI and SPIV. (**C**) Retention rate of vanillin stored at 40 °C for 14 days. * *p* ≤ 0.05, *** *p* ≤ 0.001, and “ns” indicates no significant difference.

The antioxidant activity of SPI, vanillin, and SPIV complexes was evaluated by DPPH and ABTS radical scavenging assays (Figure 7A,B). In the DPPH assay, SPIV complexes showed slightly stronger radical scavenging activity than free vanillin, which may be attributed to the intrinsic antioxidant contribution of soy protein in addition to vanillin. Since the DPPH assay was conducted in an ethanolic medium, SPIV complexes tended to disperse more uniformly or undergo partial dissociation, thereby improving the accessibility of both soy protein and vanillin to DPPH radicals. As the vanillin loading increased, the difference in antioxidant activity between SPIV and free vanillin gradually diminished, indicating that the relative contribution of soy protein became less pronounced at higher vanillin contents. In contrast, SPIV complexes and free vanillin exhibited comparable ABTS radical scavenging activity. This observation suggests that, in the aqueous ABTS system, the antioxidant contribution of soy protein was largely masked by protein–vanillin interactions, and vanillin remained the dominant radical scavenger. Therefore, the different trends observed in the DPPH and ABTS assays were likely related to the assay media. In ethanol, the complexes were more accessible to the radical, allowing soy protein to contribute to the overall antioxidant response, whereas in water, intermolecular interactions within SPIV may have hindered the direct reaction of soy protein with ABTS radicals. Such medium-dependent behavior is consistent with previous studies reporting altered antioxidant activity upon molecular complexation or encapsulation [21,36].

### 3.5. Thermal Stability of Vanillin in SPIV

Given the volatility of vanillin and the associated losses during storage and heating, the effect of SPIV complexation on vanillin stability was evaluated in terms of vanillin retention (Figure 7C). Free vanillin exhibited substantial thermal loss, which was consistent with the TGA results. In contrast, SPIV complexes effectively suppressed thermal loss of vanillin, regardless of whether it was incorporated at a high loading level in SPIV5 or at a medium loading level in SPIV2. Together with the interaction mechanisms between vanillin and SPI described above, this improved thermal stability is likely attributable to the dual role of SPI as both a wall material and a stabilizing matrix. On the one hand, SPI engages in extensive covalent and noncovalent interactions with vanillin, as described above, which reduces the tendency of vanillin to volatilize into the air [37]. On the other hand, SPI also acts as a wall material for vanillin and suppresses further thermal loss during heating. Considering the vanillin nanocrystals observed in the SEM images, this structural feature may explain why the thermal loss of SPIV5 was slightly higher than that of SPIV2.

### 3.6. Emulsifying Performance of SPIV

**Figure 8:**
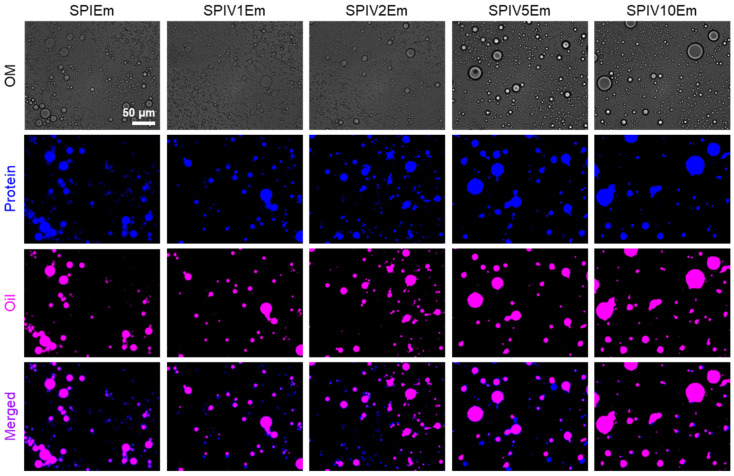
Bright-field and fluorescence micrographs of emulsions stabilized by SPI and SPIV complex. All images were acquired at identical magnifications, thus share the same scale bar, representing 50 μm.

To evaluate the practical interfacial performance of SPIV after its structural and stabilizing characteristics had been established, emulsions stabilized by SPI or SPIV were further investigated. Vanillin is an amphiphilic molecule that tends to migrate toward the oil–water interface, which often induces droplet destabilization [20,38]. Given that vanillin in SPI formed complexes, differences in interfacial tension were expected. Bright-field microscopy showed that all SPIV samples produced stable droplets, but their droplet size and dispersity differed (Figure 8). SPI formed droplets with a broad size distribution and noticeable droplet aggregation. In contrast, SPIV1 and SPIV2, with low vanillin loadings, generated more dispersed and uniform droplets. Conversely, SPIV5 and SPIV10, with high vanillin loadings, resulted in larger and less homogeneous droplets. By staining SPIV with Fast Green (blue) and oil droplets with Nile Red (pink), we observed that proteins in all groups surrounded the oil droplets. Fast Green and Nile Red were added after emulsification, so Fast Green inevitably partitioned partially into the oil phase. Nevertheless, the distinct interfacial boundary around each droplet indicates that SPIV accumulated at the droplet interface to form a shell layer, thereby stabilizing the emulsion.

**Figure 9:**
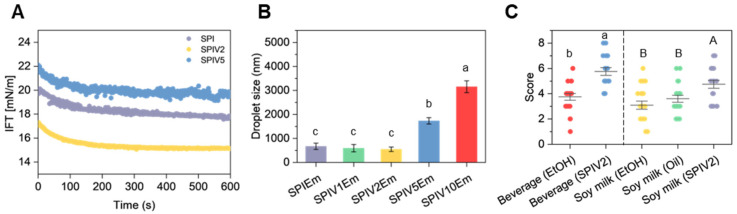
Properties of emulsions stabilized by SPI or SPIV. (**A**) Interfacial tension of SPI, SPIV2, and SPIV5 with MCT. (**B**) Droplet size of emulsion stabilized by SPI and SPIV. (**C**) Sensory evaluation of beverage and soy milk added vanillin stored in different forms stored at 40 °C for 14 days (*n* = 20). Values with different superscript letters are significantly different (*p* < 0.05).

SPI, SPIV2 and SPIV5 were selected for interfacial tension measurements to elucidate the correlation between vanillin-induced structural evolution (spherical vs. irregular morphologies) and interfacial stabilization mechanisms. The vanillin content was found to influence the microstructure and hydrophilicity of SPIV, which in turn determined the adsorption behavior of the nanoparticles at the interface [39]. As shown in Figure 9A, SPIV2, similar to SPI, induced a decrease in interfacial tension during adsorption at the interface, after which the value remained almost constant. This pattern is consistent with the typical irreversible adsorption behavior of solid nanoparticles in Pickering emulsions. In contrast, SPIV5 exhibited a distinct interfacial tension profile. Even after the initial rapid adsorption stage (<100 s), the interfacial tension of SPIV5 continued to fluctuate significantly compared to SPI and SPIV2. The interfacial stabilization at high vanillin loadings was not dominated exclusively by the SPIV complexes, and the dynamic migration of free vanillin molecules within the interfacial region may have occurred concurrently. In addition, SPIV2 retained a relatively globular morphology, whereas the globular structure of SPIV5 was disrupted, which may also contribute to differences in the interfacial distribution of SPIV complexes.

The droplet size distribution data also reflected these distinct interfacial features. SPIEm, SPIV1Em and SPIV2Em exhibited comparable droplet sizes (*p* > 0.05), whereas the droplet sizes of SPIV5Em and SPIV10Em were significantly larger (Figure 9B). During homogenization, SPIV complexes were irreversibly adsorbed onto the oil droplet surfaces. Droplets coated with these firmly adsorbed complexes exhibited improved long-term stability. SPIV5 and SPIV10 were more hydrophilic and carried crystalline vanillin on their surfaces, which may lead to reduced emulsion stability.

### 3.7. Aroma Performance in Beverage Models

Since vanillin is a widely used flavoring agent in beverage applications, SPIV2 was further evaluated in simplified soft drink and soy milk models to assess its practical flavor retention and delivery performance during storage. For comparison, free vanillin dissolved in ethanol (for soft drinks) or soy oil (for soy milk) was employed as the control group. Consistent with previous reports, accelerated storage tests revealed distinct differences in flavor stability between the SPIV2 formulations and the control samples [21]. In both beverage models, samples containing SPIV2 achieved significantly higher flavor scores than those prepared by direct addition of vanillin (Figure 9C). This improvement was likely related to the incorporation of vanillin into SPIV, which reduced thermal loss and matrix-induced flavor deterioration during storage. Notably, even when vanillin was pre-dissolved in soy oil, the corresponding flavor scores remained lower than those of SPIV2, which could be ascribed to the preferential partitioning of free vanillin into oil droplets rather than the desired aqueous phase. These results support the functional relevance of SPIV in beverage-type systems. In addition, the pH-shifting process utilized for SPIV preparation is operationally simple and relies mainly on pH adjustment and mixing, which suggests potential for scale-up. Although NaOH requires proper handling during processing, its use in food and beverage operations is already well established, which further supports the practical applicability of this strategy [40].

## 4. Conclusions

In this study, soy protein isolate–vanillin complexes (SPIV) were successfully fabricated through a pH-shifting approach, employing soy protein isolate (SPI) as both a dispersing agent and a protective matrix for vanillin. Vanillin was incorporated into SPI at loadings ranging from 9 to 50 wt.%, and the binding efficiency decreased from 91.03 wt.% to 69.43 wt.% with increasing vanillin loading. The resulting SPIV significantly enhanced the dispersibility, light stability, and thermal stability of vanillin in aqueous systems and enhanced its flavor retention in simplified beverage models. Notably, vanillin loading strongly affected the structure and functionality of SPIV. At a moderate loading level of 16.7 wt.% (SPIV2), the native globular morphology of SPI was largely preserved, and favorable interfacial behavior was obtained, whereas higher loading levels induced vanillin nanocrystal formation on the protein surface and promoted aggregation. Spectroscopic analyses together with molecular docking, with best docking scores of −5.180 and −5.755 kcal/mol, suggested that vanillin interacted with soy proteins through a combination of covalent and noncovalent interactions. Overall, this study reveals a loading-dependent structure–function relationship in soy protein–vanillin complexes, offering valuable theoretical guidance for the design of soy protein-based carriers for flavor stabilization and controlled release applications.

## Figures and Tables

**Table 1:** Free vanillin content, bound vanillin content, and vanillin binding efficiency in SPIV complexes with different vanillin contents. Values with different superscript letters in the same row are significantly different (*p* < 0.05).

	SPIV1	SPIV2	SPIV5	SPIV10
Added vanillin (mg/mL)	0.5	1	2.5	5
Free vanillin (mg/mL)	0.045 ± 0.015 ^d^	0.156 ± 0.015 ^c^	0.470 ± 0.008 ^b^	1.529 ± 0.030 ^a^
Bound vanillin (mg/mL)	0.455 ± 0.015 ^d^	0.844 ± 0.015 ^c^	2.030 ± 0.008 ^b^	3.471 ± 0.029 ^a^
Binding efficiency (wt.% bound vanillin/total vanillin)	91.03 ± 2.91 ^d^	84.43 ± 1.50 ^c^	81.20 ± 0.34 ^b^	69.43 ± 0.58 ^a^
Loading capacity (wt.% bound vanillin/SPI)	9.10 ± 0.29 ^d^	16.89 ± 0.30 ^c^	40.60 ± 0.17 ^b^	69.43 ± 0.58 ^a^

## Data Availability

The original contributions presented in the study are included in the article. Further inquiries can be directed to the corresponding author.
